# Chemical Characterisation of *Cytisus striatus*: A Multi-Technique Approach Using GC-MS, LC-HRMS/MS, NIR, and FT-RAMAN

**DOI:** 10.3390/plants15091338

**Published:** 2026-04-28

**Authors:** Débora Caramelo, Tiago A. Fernandes, Eugenia Gallardo, Ofélia Anjos, Jorge Gominho

**Affiliations:** 1 Centro de Estudos Florestais (CEF), Laboratório Associado TERRA, Instituto Superior de Agronomia, Universidade de Lisboa, 1349-017 Lisboa, Portugal; dbrcaramelo@gmail.com (D.C.); jgominho@isa.ulisboa.pt (J.G.); 2 Departamento de Ciências e Tecnologia (DCeT), Universidade Aberta, 1000-013 Lisboa, Portugal; tiago.fernandes@uab.pt; 3 MINDlab—Molecular Design & Innovation Laboratory, Centro de Química Estrutural, Institute of Molecular Sciences, Departamento de Engenharia Química, Instituto Superior Técnico, Universidade de Lisboa, 1049-001 Lisboa, Portugal; 4 RISE-Health, Departamento de Ciências Médicas, Faculdade de Ciências da Saúde, Universidade da Beira Interior, 6200-506 Covilhã, Portugal; egallardo@fcsaude.ubi.pt; 5 Laboratório de Fármaco-Toxicologia—UBIMedical, Universidade da Beira Interior, 6200-284 Covilhã, Portugal; 6 Centre for Natural Resources, Environmental and Society, Polytechnic University of Castelo Branco, (CERNAS-IPCB), 6001-909 Castelo Branco, Portugal

**Keywords:** *Fabaceae*, flavone, flavonoid, chrysin, apigenin derivatives

## Abstract

The *Cytisus striatus* species is a biologically important plant recognised for its high flavonoid content. However, the chemical composition of this species has yet to be fully described. The purpose of this study was to identify flavonoids and related secondary metabolites using advanced analytical techniques, including gas chromatography, liquid chromatography, and NIR and Raman spectroscopy. Ethanolic extracts of flowers, fruits, and twigs/leaves contain highly beneficial flavonoids, with chrysin being the main compound detected in all plant parts. It was quantified by HPLC-DAD at levels ranging from 0.90 to 2.27 mg/g extract in samples collected from three different locations. NIR and FT-RAMAN analysis provided complementary information on the overall chemical fingerprint of the plant material. PCA of the spectroscopic data revealed minor site-related differences in global spectral profiles, with PC1 explaining 85% of the variability in flowers and fruits and 72% in twigs/leaves for FT-NIR, while FT-RAMAN analysis of fruit extracts showed a PC1 accounting for 97% of the variance. This study provides a basis for future research on the biological properties of *Cytisus striatus* and for further assessment of its potential relevance in pharmaceutical and/or food-related applications.

## 1. Introduction

*Cytisus striatus* (Hill) Rothm., commonly referred to as yellow broom, is a leguminous indigenous plant to the Iberian Peninsula and included under the Fabaceae family. This shrub is noteworthy not only for its nitrogen-fixing capacity but also for its medicinal potential, having been used in traditional medicine [1,2]. In Portugal, flowers from *Cytisus striatus* and other related species have been used to treat various health problems, such as gout, rheumatic diseases, hypotension, and heart failure. These were prepared using extraction processes such as infusions and decoctions [3,4]. It is well established that flowers contain flavanols, tartaric esters, and several organic acids. Although few studies have been conducted, the existing research provides scientific support for the traditional uses of this species in folk medicine [3,5].

The flavonoid class presents a diverse group of phenolic compounds, recognised for their broad range of pharmacological activities, including antioxidant, anti-inflammatory, antimicrobial, and anticancer properties [6,7,8]. Flavonoids, such as chrysin and its derivatives, are noteworthy substances in species of the *Cytisus* genus due to their biological effects [9,10]. Chrysin is a flavone with notable antioxidant and anti-inflammatory effects, and it has been studied for its potential in modulating pathways related to oxidative stress, inflammation, and even neuroprotection. It has also been shown to have antidiabetic and anticancer properties against various cancer cell lines [11,12]. Nowadays, chrysin has been identified exclusively in methanolic extracts of *Cytisus striatus* leaves, where the study’s authors also recognise the antimicrobial potential of this broom species [10]. Although there are only a few in-depth studies on the chemical profile of the flowers, fruits, and twigs/leaves of this species. However, the fact that *Cytisus striatus* is found in many places and is important to the ecosystem suggests that further research is needed to identify bioactive compounds that could be useful in medicine and industry. Isoflavonoids, such as daidzein and genistein, frequently reported in Fabaceae species, are known for their estrogenic and antioxidant properties, highlighting the importance of a deeper phytochemical characterisation of *Cytisus striatus* to support potential pharmacological applications.

Advanced analytical techniques have become essential for elucidating and clarifying complex chemical compositions of medicinal plants, as well as those with important biological functions. Techniques like gas chromatography–mass spectrometry (GC-MS), liquid chromatography–tandem mass spectrometry (LC-MS/MS), near-infrared spectroscopy (NIR), and Raman spectroscopy provide robust tools for both qualitative and quantitative assessment [13,14]. GC-MS is extremely useful for detecting volatile compounds, while LC-MS/MS offers high sensitivity and selectivity for bioactive molecules such as flavonoids [15,16]. On the other hand, NIR and Raman spectroscopy allow for quick and non-destructive analysis of plant matrices. This provides us with complementary information that helps us understand the chemical profile of different types of samples [17].

This study offers the first comprehensive chemical profile of *Cytisus striatus*, emphasising the identification of compounds in the flowers, fruits, and twigs/leaves of the plant, as well as the specific quantification of a key flavonoid, chrysin [18]. Complementary analytical techniques with distinct functions were employed: GC-MS and liquid chromatography coupled with high-resolution electrospray ionisation mass spectrometry (LC-ESI-HRMS/MS) were used for metabolite identification and chemical profiling, whereas high-performance liquid chromatography coupled to a diode array detector (HPLC-DAD) enabled the quantification of chrysin. In parallel, FT-NIR and FT-RAMAN Raman spectroscopy provided rapid, non-destructive information on the overall chemical characteristics of the samples. The combined use of these techniques offered complementary insights into sample composition, although direct relationships between specific spectral features and individual metabolites were not established in this study. This study addresses the existing knowledge gap regarding the chemical composition of this species, applying a multi-technique approach that enables a more comprehensive and robust chemical analysis. The results provide valuable information on the chemical profile of *Cytisus striatus*, deepening our understanding of its chemical biodiversity across the different parts of the plant and supporting the identification of bioactive compounds with potential applications in pharmacology, medicine, and nutrition.

## 2. Results and Discussion

### 2.1. GC-MS Analysis

Table S1 (Supplementary Information), provided in the Supplementary Material, shows the identification of the chemical compounds in the ethanolic extracts of flowers, fruit, and twigs/leaves from the different harvesting sites. In addition, some compounds were specifically identified in flowers, fruits, and branches/leaves, which are represented in Tables S2–S4 (Supplementary Information). The majority of compounds in the flowers are essentially palmitic acid, linolenic acid, and chrysin. Meso-2,3-diphenylbutane appears in relative percentages of 10.13, 5.01, and 4.02 in the FCB, FG, and FB samples, respectively. The organic compound 5-hydroxymethylfurfural was detected in all samples, with a higher percentage in the FB sample. In the fruit, most compounds are chrysin, palmitic acid, and homopterocarpin. Oleamide also appeared with some relative percentage in the FrG (5.52%) and FrB (8.05%) samples, as did cis-vaccenic acid in the FrCB (10.43%) and FrB (9.68%) samples. On the other hand, the twigs/leaf samples showed the highest relative percentage of chrysin, TLCB (32.82%), TLG (23.91%), and TLB (18.03%). They also have palmitic acid and linolenic acid as their main compounds. The analysis of variance for identifying the different compounds revealed significant differences (*p* < 0.0015) between the various parts of the plant and harvesting sites (LxP), with values ranging from 6.8 for the compound 4H-pyran-4-one, 2,3-dihydro-3,5-dihydroxy-6-methyl to 60.8 for 2-monopalmitin. Regarding the collection location (L), there were significant differences with a range of values from −0.2 for benzoic acid to 67.2 for the alkaloid, hydroxylupanine. It should be noted that the relative peak area percentages obtained by GC–MS are semi-quantitative, as no correction for compound-specific response factors or internal standard normalisation was applied.

**Figure 1:**
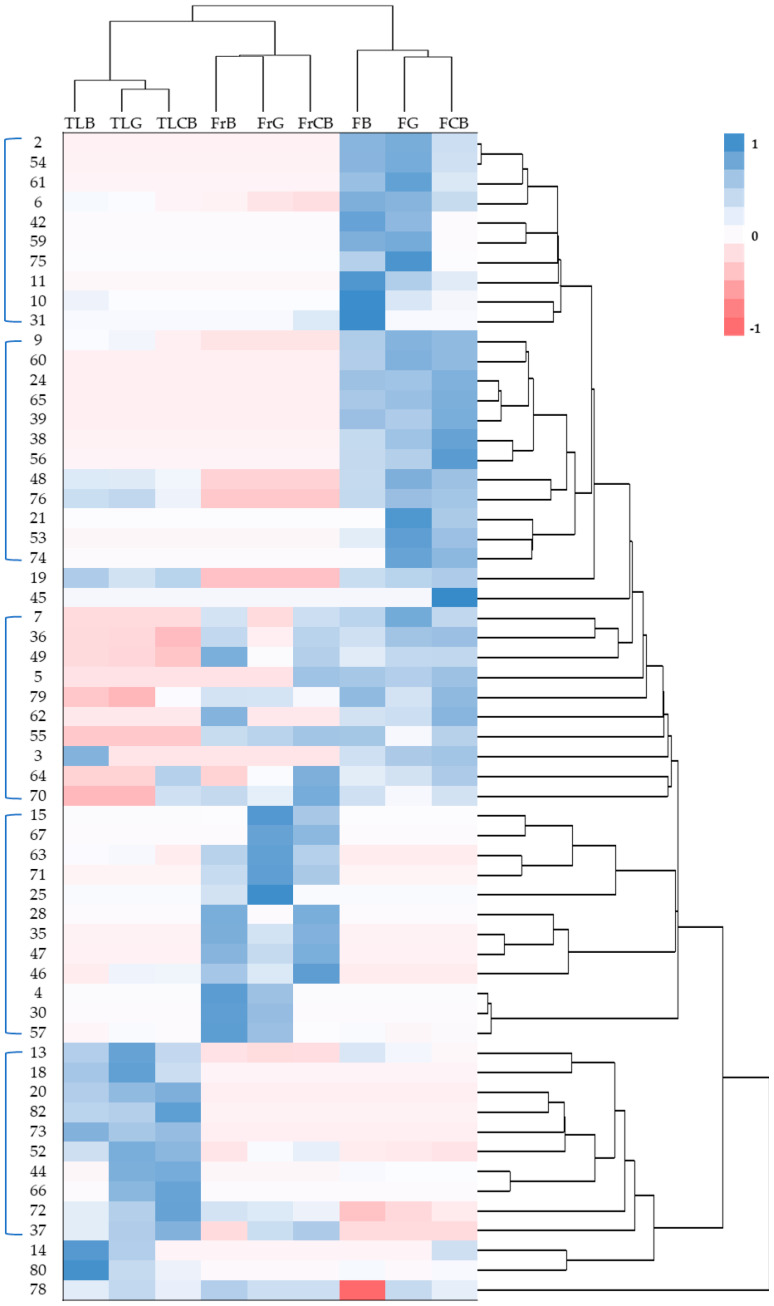
Heat map representing clusters of chemical compounds in the flowers, fruits, and twigs/leaves of *Cytisus striatus*. Legend: FCB, flowers from Castelo Branco; FG, flowers from Guarda; FB, flowers from Bragança; FrCB, fruits from Castelo Branco; FrG, fruits from Guarda; FrB, fruits from Bragança; TLCB, twigs/leaves from Castelo Branco; TLG, twigs/leaves from Guarda; TLB, twigs/leaves from Bragança; 2—furaneol, 54—pentacosane, 61—octacosane, 6—4H-pyran-4-one, 2,3-dihydro-3,5-dihydroxy-6-methyl-, 42—linolenic acid, methyl ester, 59—hexacosane, 75—hentriacontane, 11—1,2,3-propanetriol, 1-acetate, 10—5-hydroxymethylfurfural, 31—pentadecanoic acid, 9—benzofuran, 2,3-dihydro, 60—phenethyl tetradecanoate, 24—tridecanoic acid, 65—oxalic acid, 2-phenylethyl tetradecyl ester, 39—heptadecanoic acid, 38—hexadecanoic acid, ethyl ester, 56—meso-2,3-diphenylbutane, 48—linolenic acid, 76—(±)-α-tocopherol, 21—3-benzyl-4-chloro-1,2,3-triazole 1-oxide, 53—benzoyl β-d-glucoside, 74—phenethyl stearate, 19—dodecanoic acid, 45—tetrahydrorhombifoline, 7—benzoic acid, 36—palmitic acid, 49—octadecanoic acid, 5—phenylethyl alcohol, 79—β-sitosterol, 62—2-mono-palmitin, 55—3β-hydroxylupanine, 3—ethyl hydrogen malonate, 64—hydroxylupanine, 70—3β, 13α-dihydroxylupanine, 15—tetradecane, 67—medicarpin, 63—homopterocarpin, 71—pterocarpin, 25—ethyl, α-D-glucopyranoside, 28—tetradecanoic acid, 35—palmitoleic acid, 47—cis-vaccenic acid, 46—linoleic acid, 4—maltol, 30—N-acetyltyramine, 57—oleamide, 13—2-methoxy-4-vinylphenol, 18—apocynin, 20—phenol, 4-ethenyl-2,6-dimethoxy, 82—lupeol, 73—squalene, 52—lupanine, 44—phytol, 66—13α-acetoxylupanine, 72—chrysin, 37—trans-sinapyl alcohol, 14—eugenol, 80—β-amyrin, 78—stigmasterol.

Figure 1 shows the heatmap based on the GC-MS chemical profile. This shows the variation in specific metabolites identified in the ethanolic extracts of the different samples. The dendrogram (at the top of the heatmap) shows that the samples group together in a manner consistent with their locations and parts of the plant. Within each group (flowers, fruit, and twigs/leaves), the samples are grouped by Castelo Branco, Guarda, and Bragança. Some columns exhibit well-defined red and blue regions, suggesting that certain compounds may serve as chemical markers for specific parts of the plant or for individual collection sites. The top of the dendrogram shows that the samples fall into two main groups: the twig and leaf samples, and the fruit and flower samples, which cluster more closely together. The twigs/leaves have a different chemical profile from the flowers and fruit, while the fruit and flowers are more similar to each other. The twigs/leaves were shown, in an initial analysis without confirmation, to be rich in alkaloids such as lupanines and isoflavonoids, while the flowers and fruit share compounds, especially terpenoids and phenolic compounds.

Additionally, some compounds can be considered specific markers in relation to a particular part of the plant. Furaneol, for example, shows a relative percentage in the flowers and was not identified in the fruit and twig/leaf samples. On the other hand, pterocarpin was only identified in the fruit, and lupeol showed a relative percentage only in the twigs/leaves.

### 2.2. LC-ESI-HRMS/MS Analysis

The LC-ESI-HRMS/MS technique was employed for the characterisation and tentative identification of flavonols, flavones, and phenolic acid derivatives. These assignments were based on high-resolution mass data and tandem MS/MS fragment ions compared with the literature data and supported by software tools such as MZmine 4.3.0 and SIRIUS 6.0.5 [19].

**Figure 2:**
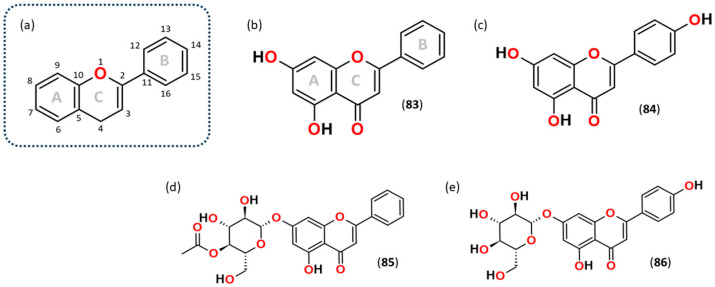
The fundamental structure of (**a**) general flavonoids, (**b**) chrysin, and (**c**) apigenin, (**d**) chrysin 7-(4″-acetylglucoside), (**e**) apigenin-7-glucoside. The rings are labelled A, B, and C, and the atoms are numbered from 1 to 16. This framework was used consistently throughout the study to facilitate easy identification of the bond or structure. Numbers (**83**), (**84**), (**85**) and (**86**) correspond to the numbering assigned to the compounds identified by LC-ESI-HRMS/MS shown in Table 1.

**Figure 3:**
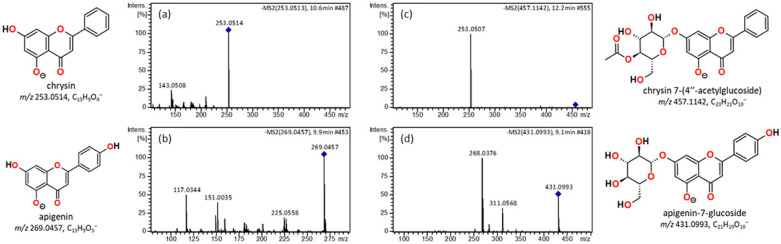
Tandem mass spectrum, in negative mode for (**a**) chrysin, (**b**) apigenin, (**c**) chrysin 7-(4″-acetylglucoside), and (**d**) apigenin-7-glucoside.

The flavones chrysin and apigenin are structurally similar; both are di-hydroxylated on the A-ring, but apigenin has one additional hydroxyl group on the B-ring (C-14) (Figure 2). Both were detected in all extracts and in both ESI modes, ESI(+) and ESI(−). The [M−H]^−^ ion (*m*/*z* 253.0514) of chrysin exhibits many fragmentation pathways, as shown in the product ion mass spectrum with *m*/*z* 143.0508 as the base peak (Figure 3, Table 1). The exhibition of many fragmentation pathways is demonstrated by a few flavonoids examined in this study. The mechanism of *m*/*z* 143.0508 can involve a ring rupture rC^3,10^, leading to the formation of a fragment ion, which is probably C_10_H_7_O^−^. The ion most certainly contains the B ring and undergoes a cyclisation rearrangement, leading to the formation of naphthalen-2-one. The other fragment ion observed for chrysin is the ring rupture rC^1,3^ leading to the low-intensity ion fragment C_8_H_7_O^−^, *m*/*z* 119.0506, 1-phenylethen-1-olate. The same exercise can be performed for the positive mode, where the [M+H]^+^ ion (*m*/*z* 255.0664) of chrysin was observed. The presence of fragment ions 153.0172 and 103.0525 is due to ring ruptures rC^1,4^A and rC^1,4^B, leaving either ring A or B intact, and giving rise to the ions C_7_H_4_O_4_^+^ and C_8_H_6_^+^, respectively, which proves the presence of chrysin. Another chrysin derivative that could have been identified was chrysin 7-(4″-acetylglucoside), which has a molecular ion of *m*/*z* 457.1142. This possible assignment is attributed to the fragment ion detected at *m*/*z* 253.0507 (C_15_H_9_O_4_^−^), which is due to deprotonated chrysin itself [chrysin − H]^−^ due to neutral loss of an acetylated hexose molecule.

Apigenin (4′,5,7-trihydroxyflavone) was identified by the presence of the [M−H]^−^ ion (*m*/*z* 269.0457), which originates the fragment ions with *m*/*z* 183.0444 and *m*/*z* 151.0032. The fragment ion *m*/*z* 183.0444 can be explained by typical retro-Diels–Alder (RDA) fragmentation and cleavage of the flavonoid C-ring, which are common fragmentation patterns, whereas the fragment *m*/*z* 151.0032 could be explained by cleavage of the C-ring between positions 1 and 3, a standard retro-Diels–Alder fragmentation mechanism found in flavone/flavonoid MS/MS fragmentation. Flavones, such as apigenin, routinely undergo C-ring fragmentation that produces a resonance-stabilised fragment, which can eliminate CO_2_ from the 4-oxo C-ring region. The result is a stable deprotonated fragment (C_14_H_9_O_3_^−^) observed at *m*/*z* 225.0558 in ESI(−). Apigenin-7-glucoside was another compound found, but only in fruit extract samples. It was identified due to the existence of the [M−H]^−^ ion *m*/*z* 431.0993. The major fragment ion *m*/*z* 268.0380, [apigenin − 2H]^•−^, corresponds to the apigenin aglycone formed by the loss of glucose (162 Da) from apigenin-7-glucoside. The fragment ion at *m*/*z* 311.0568 can be assigned as an acetylated aglycone fragment formed in the ion source. This 42 Da increase from the base aglycone indicates the presence of an acetyl group, likely existing in the gas phase as a stable 1-hydroxyethenyl enol tautomer, which acts as a diagnostic marker for the acetylation of the apigenin core. Notably, as in the LC-MS analysis, chrysin, apigenin, apigenin-7-glucoside, and chrysin 7-(4″-acetylglucoside) were found in all plant parts, including flowers, fruit, and twigs/leaves (see also Table S5 in the Supplementary Information).

The other two species, with molecular ions *m*/*z* 283.0613 and 285.0406, have an identical fragment ion with *m*/*z* 268.03. These [M−H]^−^ ions may be related to oroxylin A (observed only in fruit extracts) and kaempferol (Table 1 and Table S5). Oroxylin A (5,7-dihydroxy-6-methoxyflavone) may have been identified due to additional ion fragments. One of those with 135 Da corresponds to the B-ring fragment after RDA cleavage, which retains one hydroxy group. The methoxy group promotes neutral loss, and RDA cleavage produces the distinctive B-ring ion found in 6-methoxy flavones in negative ESI-MS. The 211 Da ion is roughly equivalent to the A-ring and a methoxy, while the fragment ion at 195 Da can be explained by a neutral loss (CH_2_O/H_2_O) from the 211 Da fragment. The possible identification of flavonol kaempferol was feasible through the identification of other ion fragments, such as 239.0355 (loss of H_2_O and CO), 199.0401 (loss of C_3_H_2_O_3_), 175.0401 (an rC^1,3^A fragment), 151 (C_7_H_3_O_4_^−^) and 133.0289 (C-ring cleavage of kaempferol). Quercetin 3-galactoside, another example from the flavonol family, can be identified by its [M−H]^−^ ion (*m*/*z* 463.0891) that originates the ion fragment with *m*/*z* 300.0277 due to the neutral loss of galactose 162 Da (C_6_H_10_O_5_) and a further loss of a hydrogen radical (H^•^, 1.0078 Da) from the deprotonated aglycone.

The isoflavones daidzin (daidzein 7-O-glucoside) and 6″-O-malonylgenistin were also attempted to be identified. Daidzin was identified by the presence of the [M+H]^+^ ion (*m*/*z* 417.1204) that originates the fragment ion with *m*/*z* 255.0667 as the base peak, corresponding to the loss of the glucoside group. The same loss was observed, in the negative mode, by the presence of the [M−H]^−^ ion (*m*/*z* 415.1029). Malonylgenistin, a glycosiloxyisoflavone that has a malonyl group instead of the hydroxy hydrogen at position 6, was another possible identification through its fragmentation. It was identified by the presence of the [M+H]^+^ ion (*m*/*z* 519.1147), which originates from the fragment ion with *m*/*z* 271.0614 as the base peak, [genistein + H]^+^, which is the protonated aglycone after genistein or malonylgenistin after loss of the entire glucose (162 Da). This is an unambiguous indication of genistein derivatives. One typical fragment of protonated genistein is the fragment ion *m*/*z* 255.0667. This is the distinctive “minor” ion of aglycone, and it is exactly comparable to the *m*/*z* 253 fragment of daidzein in negative mode. It results from either the loss of water and rearrangement or the loss of one oxygen atom (−16 Da).

The coumarin esculetin was detected by the [M−H]^−^ ion with *m*/*z* 177.0195. The thereby produced fragment ions (*m*/*z* 149.0244, 134.0364, 121.0294) are all aromatic, resonance-stabilised anions, which is why they are detected with good intensity. We were able to find the secondary fragment ion with *m*/*z* 105.0359, which is a low-mass fragment produced by further breakdown of the higher-mass coumarin fragments (*m*/*z* 149, 134 and 121 ions) even more. Esculetin has two phenolic -OH groups and a lactone carbonyl. The deprotonation may occur on a phenolic -OH with the negative charge being delocalised across the aromatic system and carbonyl. That delocalisation makes it easier for the CO elimination, lactone opening, and rearrangements that assist the loss of small neutral molecules (CO, CH_2_O, etc.).

Two phenolic acids may also be identified: trans-O-coumaric acid 2-glucoside with [M−H]^−^ ion *m*/*z* 325.0935 and 2-cinnamoyl-1-galloylglucose with [M−H]^−^ ion *m*/*z* 461.1092. The fragment ions *m*/*z* 163.0397 (loss of the glucose yielding the deprotonated coumaric-acid aglycone) and *m*/*z* 119.0503 (resulting from the decarboxylation of that aglycone) aid in the identification of trans-O-coumaric acid 2-glucoside. The compound tentatively identified as 2-cinnamoyl-1-galloylglucose yielded a fragment ion at *m*/*z* 253.0503, which is proposed to arise from the loss of glucose and small neutrals or by intramolecular acyl transfer. This fragment is consistent with the hypothesised resonance-stabilised deprotonated acyl-acyl fragment, corresponding to the ion C_15_H_9_O_4_^−^. Interestingly, quercetin 3-galactoside, esculetin and trans-O-coumaric acid 2-glucoside were only detected in the flowers and may function as markers for this source of extracts.

Although heatmap analysis was applied to the GC–MS dataset as an exploratory approach to compare global volatile profiles, the LC–MS/MS results were treated in a targeted manner. Nevertheless, multivariate analysis of the LC–MS/MS dataset may provide additional insight into sample clustering and should be explored in future studies.

Notably, as in the GC-MS analysis, chrysin was detected in all plant parts, including the flowers, fruit, and twigs/leaves. LC-MS/MS analysis revealed distinct phenolic profiles among the plant organs (Table S5). The genera *Genista* and *Cytisus*, including *Cytisus scoparious*, *Cytisus multiflorus* and *C. striatus*, are rich in flavonoids, such as apigenin, chrysin, and other flavones and flavonols, as well as isoflavonoids, with profiles varying by plant organ and phenological stage. Isoflavones, such as daidzein and genistein, are particularly abundant and contribute to diverse biological activities, including antioxidant, anti-inflammatory, hypoglycaemic, estrogenic, and cytotoxic effects [20]. Detailed phytochemical analyses of *C. multiflorus* flower extracts using ESI-MS and NMR identified chrysin-7-O-D-glucopyranoside as the main compound, alongside rutin, luteolin derivatives, and other uncommon flavonoids [9]. In *Cytisus scoparious* seed pods, orientin was the predominant flavone, while the flowers were rich in rutin, quercetin, and isoquercetin. Kaempferol was detected exclusively in the pods, highlighting the organ-specific variation in flavonoid composition [21]. Compared to our work, kaempferol was identified in all parts of the plant, as were apigenin and quercetin derivatives. However, rutin and luteolin derivatives were not identified in the species selected for this study.

On the other hand, Abreu *et al.* reported that a bioactive fraction isolated from methanolic extracts of *Cytisus striatus* leaves contained several isoflavonoids, including apigenin, chrysin, daidzein, genistein, as well as hydroxylated derivatives such as 2′-hydroxygenistein and 3′-hydroxydaidzein [10]. Conversely, an additional study examined ethanolic extracts obtained from *C. striatus* flowers harvested in Spain [22]. Using LC-HRMS/MS, a total of 27 phenolic compounds were identified, with chrysin being one of the major constituents, together with quercetin and apigenin derivatives such as quercetin 3-galactoside and apigenin-7-glucoside. Although these findings emphasise the antioxidant and anti-inflammatory properties of the species, the study did not elucidate the distribution of these metabolites across different plant organs. In the present work, beyond chrysin, the identification of flavones (apigenin, apigenin-7-glucoside, chrysin 7-(4″-acetylglucoside)), flavonols (kaempferol, quercetin 3-galactoside), flavones (daidzein 7-O-glucoside, malonylgenistin), and phenolic acid derivatives (trans-O-coumaric acid 2-glucoside, 2-cinnamoyl-1-galloylglucose) and the coumarin esculetin is particularly relevant, as these compounds are recognised for their antioxidant, estrogenic, and chemopreventive properties. Their occurrence further supports the pharmacological value of *C. striatus* as a promising source of bioactive metabolites.

### 2.3. HPLC-DAD Analysis

**Figure 4:**
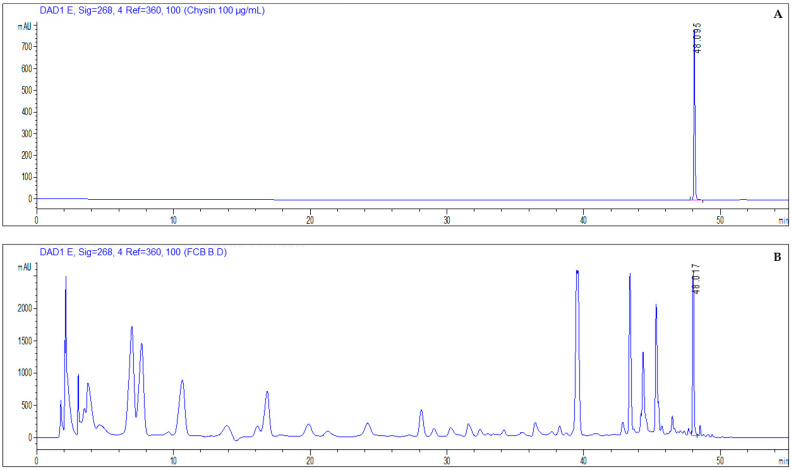
Chromatograms obtained by HPLC-DAD, where (**A**) is the chrysin standard and (**B**) is a sample analyzed, in this case, flowers collected in Castelo Branco.

Based on its detection by LC-ESI-HRMS/MS and GC-MS in all plant parts, chrysin was selected for subsequent quantification by HPLC-DAD (Figure 4). Table 2 summarises the chrysin quantified in the different samples of flowers, fruit, and twigs/leaves from the selected species, along with the wavelength and retention time of this flavonoid. Chrysin (5,7-dihydroxy-2-phenyl-4H-chromen-4-one) is a polyphenol of the flavone class with a natural 15-carbon skeleton. Chrysin comes from a few natural sources, such as honey, propolis, and passion fruit (*Passiflora* spp.). Scientific studies have shown that this chemical has neuroprotective and hepatoprotective effects, as well as promotes reproductive health, as observed in various animal models [11]. In this work, it was observed that, in general, the flowers of the yellow broom contain values between 1.72 and 1.78 mg of chrysin/g of extract. The fruits have a chrysin content of between 1.35 and 2.27 mg/g, and the twigs/leaves between 0.90 and 1.48 mg/g. Considering all parts of the plant, the flowers and fruit have the highest chrysin content. In a study carried out by Pereira et al. [9], ethanolic extracts from the flowers of *Cytisus multiflorus*—a species very similar to the yellow broom—were also evaluated for their chrysin content by HPLC-DAD. The authors in this study found lower chrysin values than those in our study (0.5 mg/g).

On the other hand, other authors have used micellar liquid chromatography on an RP-HPLC to determine the amount of chrysin in honey. They obtained values of 0.48 mg/kg of chrysin [23]. Recently, this flavonoid was quantified in samples of leaves, green and ripe pulp, and green and ripe peel of the *Passiflora caerulea* species, using HPLC-DAD and the QuEChERS extraction method. In this study, the levels of chrysin found in the leaf were 5 mg/kg, and in the green peel were 0.35 mg/kg. The green and ripe pulp, as well as the ripe peel, showed even lower levels of chrysin than the other parts of the plant studied [24].

### 2.4. FT-NIR and FT-Raman Spectral Analysis

**Figure 5:**
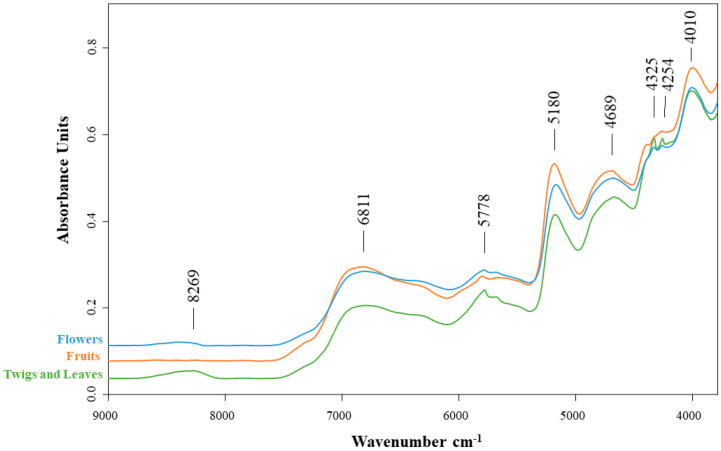
FT-NIR spectra of flowers, fruits, and twigs/leaves of *Cytisus striatus*.

A representative spectrum of a sample of flowers, fruit, and twigs/leaves of *Cytisus striatus* before extraction (powder only) obtained by FT-NIR is shown in Figure 5.

The NIR spectrum shows weak absorption in the 9000–8000 cm^−1^ region, where the vibrational band at 8269 cm^−1^ may be associated with C-H stretching overtones. The region of more pronounced bands, namely at 6811 cm^−1^ and 5778 cm^−1^, is generally related to combinations of O-H and N-H stretching vibrations, which may reflect contributions from phenolic and other secondary metabolites [25]. The most intense region is represented by the 5500–4000 cm^−1^ region, which is typically characterised by the absorption of water and organic compounds. The 5180 cm^−1^ peak, which shows a significant intensity in all samples, is attributed to O-H combinations and the first overtones of water and ethanol, which may contribute significantly to the overall spectral variability [26]. However, as all samples were prepared using the same extraction protocol and solvent composition, this band represents a consistent spectral background throughout the dataset. Solvent-related signals of this kind are retained in PCA, but they do not contribute to sample discrimination, although they may influence the orientation and scaling of the principal components. The separation observed in PCA score plots is therefore primarily associated with differences in the biochemical composition of the samples, particularly variations in phenolic compounds and other metabolites.

The remaining vibrational bands, 4689 cm^−1^, 4325 cm^−1^, 4254 cm^−1^, and 4010 cm^−1^, may be associated with C-H contributions characteristic of secondary metabolites such as flavonoids [27].

The fruit samples exhibit more intense absorption at 5180 cm^−1^, which could mean they have a higher content of hydroxylated compounds or water than the flower and twigs/leaves samples. The twigs and leaves exhibit lower overall absorption but have two more intense bands than the other samples (at 4325 cm^−1^ and 4254 cm^−1^). The absorbance of the flowers resembles that of the fruit from various places, displaying an intermediate pattern.

**Figure 6:**
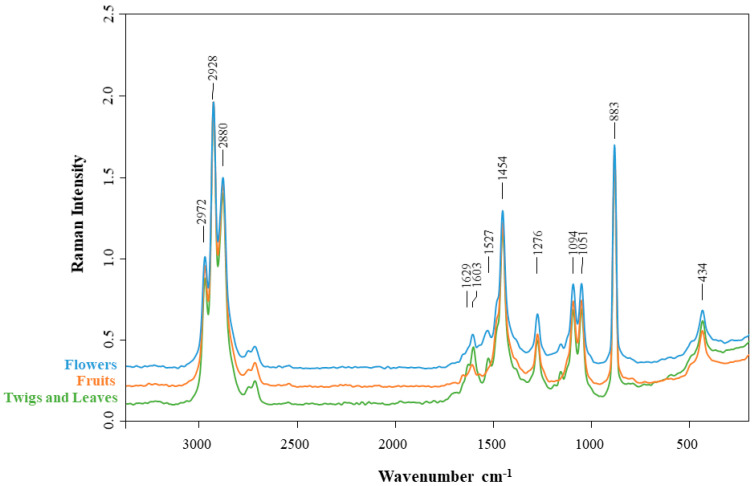
FT-Raman spectra of ethanolic extracts of flowers, fruits and twigs/leaves of *Cytisus striatus*.

Raman spectroscopy is a powerful vibrational technique for identifying the unique fingerprint signatures of different compounds. Figure 6 shows the FT-Raman spectra of ethanolic extracts from flowers, fruits, and twigs/leaves of the *C. striatus* species, which highlights the significant bands in all plant parts.

All the spectra show a strong and typical band at approximately 2928 cm^−1^. This band can be attributed to the asymmetric C-H stretching vibration of saturated hydrocarbons and hydrocarbon substituent groups [17,28,29,30]. The spectra are particularly different in the range of 1600 cm^−1^, where these bands are associated with the C=C stretching vibrations of the aromatic ring at 1606 cm^−1^ and 1528 cm^−1^ [31]. The vibrational bands at 1606 cm^−1^ may originate from the C=C bond, or the aromatic ring (C-C) present in lignin derivatives from plant cell walls [32]. So, the spectrum of twigs and leaves, which has a stronger band than that of flowers and fruit, may explain this. The band at 1528 cm^−1^ might possibly be affected by the N-H bending vibrations of primary and secondary amines, which are commonly observed in this region of the spectrum [17,26,27]. The protein content in the flowers and fruit of *C. striatus*, as identified in a previous study carried out by our group, could explain this [33].

Upon analysing the spectra in the wavelength range from 1550 to 950 cm^−1^, several peaks were identified with minimal variation between the different parts of the plant. The vibrational band at 1454 cm^−1^ is associated with the C-H bending and C=C-C stretching modes of aromatic structures. The high-intensity peak at 883 cm^−1^ is attributed to glycosidic C–O–C vibrations typical of carbohydrate structures, including polysaccharides such as cellulose and related compounds [17]. Given the inherent complexity of plant matrices, band assignments obtained from FT-Raman analysis should be regarded as tentative.

**Figure 7:**
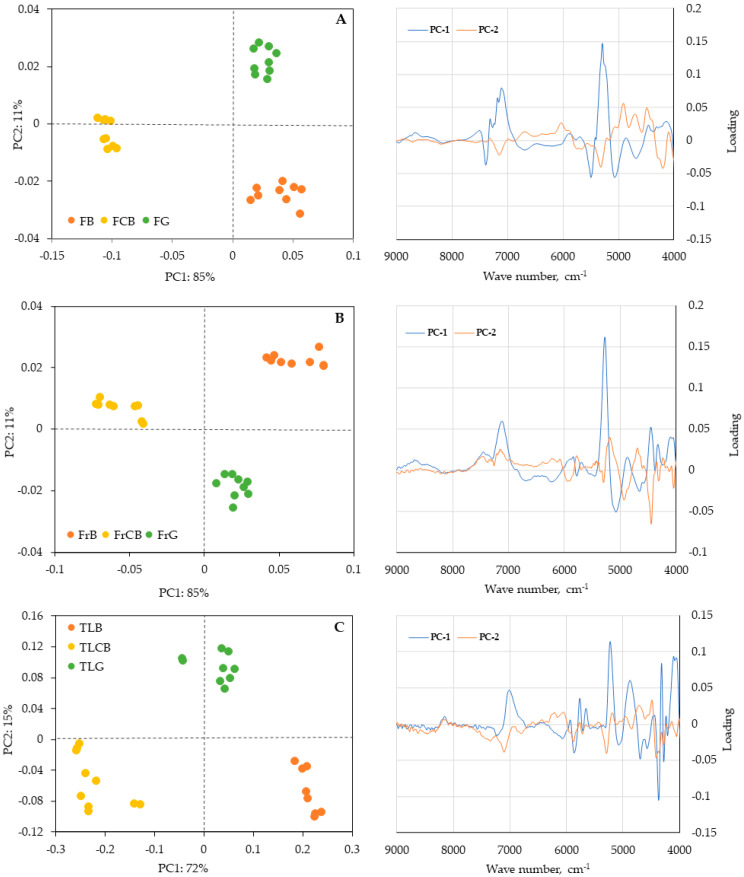
Principal component analysis and their corresponding loadings plots, of samples of flowers, fruits, and twigs/leaves of *Cytisus striatus* in three different sites for: (**A**) NIR spectral information scores of flowers; (**B**) NIR spectral information scores of fruits; (**C**) NIR spectral information scores of twigs/leaves. Labels: FCB, flowers from Castelo Branco; FG, flowers from Guarda; FB, flowers from Bragança; FrCB, fruits from Castelo Branco; FrG, fruits from Guarda; FrB, fruits from Bragança; TLCB, twigs/leaves from Castelo Branco; TLG, twigs/leaves from Guarda; TLB, twigs/leaves from Bragança.

**Figure 8:**
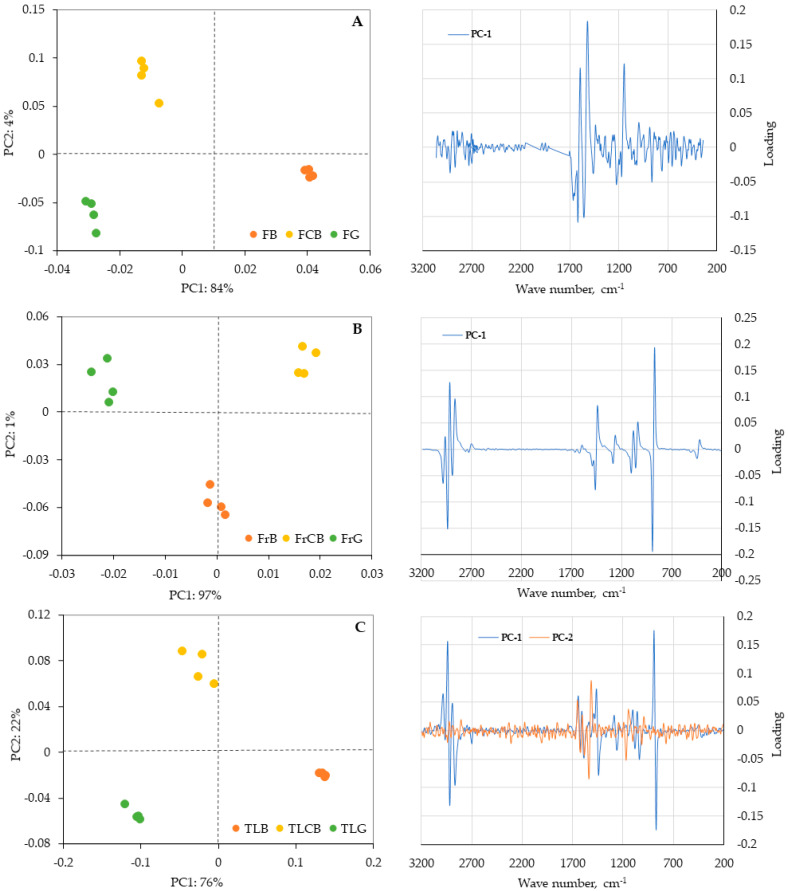
Principal component analysis and their corresponding loadings plots, of samples of flowers, fruits, and twigs/leaves of *Cytisus striatus* in three different localities for: (**A**) FT-Raman spectral information scores of flowers; (**B**) FT-Raman spectral information scores of fruits; (**C**) FT-Raman spectral information scores of twigs/leaves. Labels: FCB, flowers from Castelo Branco; FG, flowers from Guarda; FB, flowers from Bragança; FrCB, fruits from Castelo Branco; FrG, fruits from Guarda; FrB, fruits from Bragança; TLCB, twigs/leaves from Castelo Branco; TLG, twigs/leaves from Guarda; TLB, twigs/leaves from Bragança.

Principal component analysis (PCA) was employed to identify variations among samples of the *C. striatus* species collected from different locations (Castelo Branco, Guarda, and Bragança) through qualitative analysis (Figure 7 and Figure 8). This mean-centred PCA was performed using spectral data obtained by FT-NIR and FT-RAMAN spectroscopy, applying the first derivative of the Savitzky–Golay algorithm with 17 smoothing points, followed by singular value decomposition (SVD). The model evaluation was performed using a random cross-validation method using 20 segments. As PCA is an unsupervised exploratory method, this procedure does not imply predictive model validation but was used to assess the stability of the data structure. PCA was conducted separately for each plant part to preserve sample homogeneity, considering the inherent variability between matrices, enabling the evaluation of potential differences between sampling locations within each plant part.

The results presented in Figure 7 show that the different samples of *C. striatus* (without extraction) from each site can be clearly distinguished from each other, suggesting that the sites exhibit minor variations in the overall composition of plant material. PC1 accounts 85% of the variability in the flowers and fruit, and 72% of the variability in the twigs/leaves. The loading plots reveal that PC1 is primarily determined by O–H overtone and combination bands (7000–5000 cm^−1^), which indicates that overall matrix effects such as moisture content and hydrogen bonding significantly impact sample separation. Phenolic compounds, which exhibit weaker NIR bands mainly related to aromatic C–H overtones and O–H combination modes (6200–4200 cm^−1^), also influence the variance but not as much. Their impact becomes more noticeable in PC2, where subtle chemical differences between treatments are apparent. Therefore, both matrix effects and phenolic composition play roles in the resulting clustering.

Therefore, FT-Raman spectroscopy may be a valuable technique for monitoring overall compositional variation among broom flowers, fruits, and twigs/leaves. The PCA results presented in Figure 8 show that, as with NIR, the samples from each site are well separated. In this case, the ethanolic extracts were analysed, which may explain why the separation of the sites is more pronounced than in the NIR. The fruits show a PC1 equal to 97% of the variance, indicating a strong separation between the sites. In the Raman data, the PC1 loadings are mainly influenced by strong bands between 1600 and 1000 cm^−1^, which can be attributed to C–C and C=C vibrations in the aromatic rings of carbohydrate and other matrix components. This indicates that the primary separation in the score plots is primarily driven by these broad structural features of the sample matrix. However, bands related to phenolic structures within this range also affect the variance, suggesting that variations in phenolic composition are important factors in distinguishing the samples.

Taken together, the spectroscopic and chromatographic results provided complementary information. While the spectroscopic techniques captured the overall chemical profile of the extracts, GC-MS revealed differences in the volatile/semi-volatile fraction, and LC-ESI-HRMS/MS enabled a more detailed characterisation of the phenolic fraction. This combined approach supported a broader interpretation of compositional differences among plant parts and collection sites. However, direct assignment of specific spectral features to individual metabolites was beyond the scope of this study, and such associations should be regarded as tentative.

## 3. Materials and Methods

### 3.1. Chemicals

For LC-ESI-HRMS/MS analysis, standard solutions were prepared using a 75:25 (*v*/*v*) mixture of ethanol and water. All solvents used in the chromatographic analysis, including ethanol, water, and formic acid, were of LC-MS grade and obtained from Sigma-Aldrich (St. Louis, MO, USA). The chrysin standard used in the diverse analysis was also obtained from Sigma-Aldrich (St. Louis, MO, USA).

### 3.2. Sample Preparation

Samples of flowers, fruits, and twigs/leaves were gathered from three separate locations in Portugal: Escola Superior Agrária in Castelo Branco (39°49′36.2″ N 7°27′44.0″ W), Vila Fernando in Guarda (40°29′27.5″ N 7°10′04.6″ W), and Montesinho Natural Park in Bragança (41°56′13.1″ N 6°37′46.4″ W). All the material collected was left to air dry at room temperature until it was thoroughly dry. The flowers and fruit were then crushed using a crusher. The twigs, along with the leaves, were ground in a blade mill until the samples were reduced to powder. The branch/leaf samples were then sieved to obtain a particle size in the 40–60 mesh fraction. All the material was stored in a dark place until the extraction process.

### 3.3. Extraction Procedure

For the different analysis techniques, 1 g of the sample was extracted with 10 mL of ethanol (99%) in an orbital plate shaker for 24 h with constant shaking. Then, all the samples were filtered, and one replicate of each extraction was obtained. For each site and plant part, samples were collected from three different plants from the same location (*n* = 3 biological replicates). Each biological replicate was extracted separately, and each extract was analysed in triplicate to assess analytical repeatability.

### 3.4. GC-MS Analysis

The volatile profile of the flower, fruit, and branch/leaf extracts was analysed in triplicate using gas chromatography coupled with mass spectrometry (GC/MS SCION-SQ 456 GC, Bruker, Fremont, CA, USA) equipped with a HP-5MS capillary column (30 m × 0.25 mm × 0.25 µm). The carrier gas used was helium at a flow rate of 1 mL/min. The initial oven temperature was set at 50 °C, gradually increasing by 7 °C/min to 160 °C, with a hold time of 2 min, and finally increasing to 280 °C with a heating rate of 4 °C/min, maintaining this final temperature for 14 min. The injector and detector were kept at 220 °C and 250 °C, respectively. The flower (100 mg/mL) and branch/leaf (100 mg/mL) samples were injected with a volume of 1 µL, in triplicate, using a split ratio of 1:50, except for the fruit (50 mg/mL) samples, which were injected with a volume of 2 µL. Compound identification was performed by comparing the obtained mass spectra with those from the NIST mass spectral library (NIST 17), considering matches with a similarity score higher than 80%. When available, identification was further confirmed using commercial reference standards. In addition, mass spectra were manually inspected to verify consistency between the experimental fragmentation pattern and the proposed compound. Only well-resolved peaks were considered in order to minimise the influence of potential co-eluting compounds. Retention indices were not determined; therefore, identifications not confirmed with standards should be considered tentative. Chrysin was identified in the GC-MS analysis by comparison with an authentic standard analysed under identical conditions, based on matching retention time and EI mass spectrum; no derivatization was applied. Its presence was further corroborated by LC-ESI-HRMS/MS. The relative amount of each compound was determined using a semi-quantitative approach based on relative peak areas, expressed as the percentage of the individual compound’s peak area relative to the total integrated peak area of all identified compounds in the samples.

### 3.5. LC-ESI-HRMS/MS Analysis

The ethanolic extracts were analysed using an Elute UPLC system (Bruker, Bremen, Germany) coupled to a Bruker Impact II quadrupole time-of-flight mass spectrometer with an electrospray ionisation (ESI) source (Bruker Daltoniks, Bremen, Germany). The methodology used to identify the representative compounds was previously developed [34]. The chromatographic separation was performed using a Luna C18 column (3.0 µm, 2.0 × 150 mm) from Phenomenex. The experiment used a flow rate of 170 µL/min, and the mobile phase consisted of two components: 0.1% formic acid in water (‘mobile phase A’) and acetonitrile with 0.1% formic acid (‘mobile phase B’). The elution programme followed a specific sequence: starting from 5% to 50% B for 6 min, switching from 50 to 100% B for 4 min, maintaining an isocratic elution with 100% B for 5 min, followed by a gradient from 100 to 5% B for 4 min and finishing with 5% B for 9 min. Before analysis, the samples were subjected to filtration without further preparation using a sterile syringe filter fitted with a hydrophilic PVDF membrane of 0.45 μm pore size and 25 mm diameter. The injection volume was 10 μL. Temperature control was maintained at 8 °C for the autosampler and 40 °C for the column. Spectra were acquired in positive and negative electrospray ionisation modes. The mass spectrometry parameters used were as follows: end plate offset set at 500 V; capillary voltage at (±) 4.5 kV; nebuliser pressure maintained at 40 psi; dry nitrogen flow rate set at 8 L/min, and heater temperature maintained at 200 °C. Internal calibration utilised sodium formate agglomerate in high-precision calibration (HPC) mode. Data acquisition covered the *m*/*z* 50–1000 range in data-dependent MS/MS mode, with an isolation window of 0.5, an acquisition rate of 3 Hz, and a fixed cycle length of 3 s. Precursor ions that satisfied an absolute threshold of 153 were selected for automatic MS/MS, with the active exclusion mode set to three spectra and released after 1 minute. The instrument operates in data-dependent acquisition mode, in which it only fragments ions with an intensity above 153 units. Nevertheless, precursor ions with intensities up to five times higher than those previously observed were also examined.

MZmine’s MZWizard was used to process the data to ensure the workflow’s reproducibility [35]. For SIRIUS 6.0.5., molecular formulas were assigned using Sirius scores with a significance threshold of 0.85 [19]. Structural annotations via CSI:FingerID were filtered based on the Tanimoto similarity score between the predicted and experimental fragmentation fingerprints. Only hits achieving a Tanimoto similarity of >0.85 were considered. Candidates were only accepted if there was a clear separation in scores between the top-ranked structure and the subsequent runner-up, minimising ambiguity in isomer selection. All significant features discussed in the results were manually validated by inspecting SIRIUS’s fragmentation trees to ensure that the major MS2 fragments matched plausible substructure losses. Mirror plots were confirmed to make sure that the experimental spectra matched the library standards in terms of both intensity and *m*/*z* accuracy.

### 3.6. HPLC-DAD Analysis

The chrysin standard solution was diluted with ethanol to a final concentration of 1 mg/mL and stored at 4 °C in the dark. A high-performance liquid chromatography system with a diode array detector (HPLC-DAD) from Agilent Technologies was used to analyse the samples. The samples, which had already been extracted with ethanol, were filtered through a 0.22 µm cellulose acetate syringe filter before being injected into the chromatographic system. The chromatographic separation took place on a YMC-Triart PFP (150 × 4.6 mm, 5 µm i.d.) with pre-column (Solitica, Arruda dos Vinhos, Portugal). The mobile phase comprised acetonitrile (A) and 0.1% trifluoroacetic acid in water (B) in a gradient mode: starting with 10% A (0–3 min), 10–15% A (3–15 min), 15% A (15–20 min), 15–18% A (20–25 min), 18–30% A (25–40 min), 30–50% A (40–45 min), and 50–100% A (45–50 min), before returning to 10% A (50–55 min). The flow rate was 1 mL/min, with an injection volume of 50 µL. The temperatures of the column and the sampler were kept at 35 °C and 4 °C, respectively. The analytes detected exhibited absorbance in the wavelength range of 255 to 532 nm. Chrysin was detected at 268 nm, as identified by comparing its retention time (tR: 48.01 min) with that of the analysed analytical standard. Calibration curves were constructed using seven concentration levels of chrysin standard solution (1.56, 3.13, 6.25, 12.50, 25, 50, and 100 µg/mL), each analysed in quintuplicate, and a new calibration curve was prepared on each analytical day. A representative regression equation was y = 204.02x − 28.376 with R^2^ = 0.9971, indicating satisfactory linearity across the studied range. The lowest validated concentration level was established as the LLOQ, based on replicate analysis and analytical performance. Six replicate injections at this level provided a coefficient of variation (CV) of 17.34%, a relative error (RE) of ±16.89%, and a signal-to-noise ratio of 5, supporting its use as the lowest quantifiable concentration. This value was 1.56 µg/mL. As the limit of detection was not systematically investigated, the concentration corresponding to the LLOQ was conservatively considered the method’s practical detection limit under the present experimental conditions, since it provided a signal-to-noise ratio greater than 3. Method precision and accuracy were assessed at four concentration levels (low, low/medium, medium, and high). Intra-day performance was evaluated using five replicates at each level within a single day, while inter-day performance was assessed over five consecutive days using three replicates per level per day. As shown in Table 3, CV values were below 15% and RE values remained within ±13% at all concentration levels, except at the LLOQ, where acceptance criteria allow a coefficient of variation of ≤20% and a RE of ±20% [36].

### 3.7. Vibrational Spectroscopy

Dried powder samples of each part of the *Cytisus striatus* species (flowers, fruits, and twigs/leaves) were analysed in a NIR spectrometer (MPA Bruker) in transmitted light mode. Each sample was analysed nine times, with 64 scans per spectrum and a spectral resolution of 16 cm^−1^ in the spectral region from 3700 to 7500 cm^−1^. A background was made between each sample. The methodology outlined in a previous study [37] was used to obtain the spectra of the extracts from *C. striatus* flowers, fruits, and twigs/leaves. For this purpose, an FT-Raman spectrometer (BRUKER, MultiRAM, Bruker Portugal Unipessoal, Lisbon, Portugal) was employed, with a high-performance 180° collecting lens, an ultra-sensitive Ge diode detector cooled with liquid nitrogen, and an integrated 1064 nm Nd:YAG laser pumped by diodes with a maximum output power of 500 mW. The spectral acquisition parameters included 50 scans per spectrum, a spectral resolution of 32 cm^−1^, a scanning speed of 5 kHz, and a wavenumber range of 4000 to 200 cm^−1^. The spectra were obtained from the ethanolic extract of each plant part studied. Measurements were made in triplicate using an 8 mm quartz optical cell with a mirrored opposite face.

### 3.8. Data Analysis

The data obtained from the GC-MS analysis were subjected to statistical analysis using a factorial analysis of variance (ANOVA) to identify statistically significant differences between the parameters measured in the various samples. The variables used were as follows: location of the plant harvested and part of the plant (flowers, fruits and twigs/leaves). The least significant difference (LSD) test was applied within the ANOVA framework to determine whether individual means differed significantly from one another. The heatmap and dendrogram were generated using Pearson correlation, and the distance metric and hierarchical clustering with complete linkage. The PCA was performed with spectral data, which was used as an unsupervised exploratory tool. The analysis was conducted using STATISTICA 7 software (StatSoft Inc., Tulsa, OK, USA). For spectral data analysis, OPUS^®^ version 7.5.18 (Bruker Optics, Ettlingen, Germany) and UnscramblerX 10.5 (CAMO, Oslo, Norway) were utilised.

## 4. Conclusions

This study presents, for the first time, an evaluation of the chemical profile of the different fractions of *Cytisus striatus* (flowers, fruits, and twigs/leaves). In addition, the species was characterised in three different locations across Portugal. The application of diverse analytical techniques played a crucial role in identifying the various classes of compounds that define this species. Moreover, the quantification of chrysin in different parts of the plant was achieved quickly and efficiently using the HPLC-DAD technique. Understanding the chemical profile of *Cytisus striatus* contributes to the phytochemical knowledge of this species and may support future comparative studies within the Fabaceae cultivars. In addition to chrysin, apigenin and related glucosides, isoflavonoids, such as daidzein and 6″-O-malonylgenistin, were also detected, highlighting the chemical diversity of the species and justifying future investigation into its potential biological relevance. Although these compounds have been associated in the literature with estrogenic and antioxidant activities, their presence alone does not demonstrate biological efficacy. Therefore, this study should be regarded as a chemical profiling survey, and further work is needed to isolate these compounds and evaluate their bioactivity, bioavailability and pharmacological relevance.

## Figures and Tables

**Table 1:** Flavone, flavonol, isoflavone, coumarin and phenolic acid derivatives tentatively identified by LC-ESI-HRMS/MS in extracts from flowers, fruits, and twigs/leaves of *Cytisus striatus*.

	Compound (ref)	RT * (min)	Molecular Formula	Adduct	*m*/*z* (exp)	Error (ppm)	Fragment Ions *m*/*z* (Error ppm, Molecular Formula)
**Flavone**	Chrysin (5,7-dihydroxyflavone) (**83**)	10.6	C_15_H_10_O_4_	[M−H]^−^	253.0514	3.0	143.0508 (3.93, C_10_H_7_O^−^) 119.0506 (3.04, C_8_H_7_O^−^)
	Apigenin (4′,5,7-trihydroxyflavone) (**84**)	9.9	C_15_H_10_O_5_	[M−H]^−^	269.0457	0.6	225.0558 (0.3, C_14_H_9_O_3_^−^) 183.0444 (−4.1, C_12_H_7_O_2_^−^) 151.0032 (−3.2, C_7_H_3_O_4_^−^)
	Chrysin 7-(4″-acetylglucoside) (**85**)	12.2	C_23_H_22_O_10_	[M−H]^−^	457.1142	0.4	253.0507 (0.3, C_15_H_9_O_4_^−^)
	Apigenin-7-glucoside (**86**)	9.1	C_21_H_20_O_10_	[M−H]^−^	431.0993	1.9	311.0568 (2.2, C_17_H_11_O_6_^−^) 268.0380 (−1.0, C_15_H_8_O_5_^•−^)
	Oroxylin A (5,7-dihydroxy-6-methoxyflavone) (**87**)	10.1	C_16_H_12_O_5_	[M−H]^−^	283.0613	0.4	268.0369 (−5.1, C_15_H_8_O_5_^•−^) 239.0341 (−3.7, C_14_H_7_O_4_^−^) 211.0400 (−0.3, C_13_H_7_O_3_^−^) 195.0457 (2.8, C_13_H_7_O_2_^−^)
**Flavonol**	Kaempferol (3,4′,5,7-tetrahydroxyflavone) (**88**)	9.7	C_15_H_10_O_6_	[M−H]^−^	285.0406	0.5	268.0380 (−1.0, C_15_H_8_O_5_^•−^) 239.0355 (2.2, C_14_H_7_O_4_^−^) 199.0401 (0.2, C_12_H_7_O_3_^−^) 175.0401 (0.2, C_8_H_5_O_2_^−^) 151.0034 (−1.8, C_7_H_3_O_4_^−^) 133.0289 (−4.5, C_8_H_5_O_2_^−^)
	Quercetin 3-galactoside (**89**)	9.0	C_21_H_20_O_12_	[M−H]^−^	463.0891	1.9	300.0277 (−1.3, C_15_H_8_O_7_^•−^)
**Isoflavone**	Daidzin (Daidzein 7-*O*-glucoside) (**90**)	9.8	C_21_H_20_O_9_	[M−H]^−^	415.1029	−1.3	253.0505 (−0.52, C_15_H_9_O_4_^−^)
	6″-*O*-Malonylgenistin (**91**)	8.7	C_24_H_22_O_13_	[M+H]^+^	519.1147	2.6	271.0614 (4.8, C_15_H_10_O_5_^+^) 255.0667 (5.9, C_15_H_10_O_4_^+^)
**Coumarin**	Esculetin (6,7-dihydroxycoumarin) (**92**)	9.2	C_9_H_6_O_4_	[M−H]^−^	177.0195	0.9	149.0244 (−0.1, C_8_H_5_O_3_^−^) 134.0364 (−6.9, C_8_H_6_O_2_^−^) 121.0294 (−0.9, C_7_H_5_O_2_^−^) 105.0359 (7.3, C_7_H_5_O^•−^)
**Phenolic acid**	*trans*-*O*-Coumaric acid 2-glucoside (**93**)	9.0	C_15_H_18_O_8_	[M−H]^−^	325.0935	1.9	163.0397 (−2.3, C_9_H_7_O_3_^−^) 119.0503 (0.5, C_8_H_7_O^−^)
	2-Cinnamoyl-1-galloylglucose (**94**)	9.4	C_22_H_22_O_11_	[M−H]^−^	461.1092	0.6	253.0503 (−1.3, C_15_H_9_O_4_^−^)

* Retention Time.

**Table 2:** Quantification of chrysin in the ethanolic extracts from the three different parts of the *Cytisus striatus* species by HPLC-DAD using analytical standard and calibration curve.

Part of Plant	(mg of Chrysin)/(g of Extract)		
	Castelo Branco	Guarda	Bragança
Flowers	1.72	1.78	1.75
Fruits	1.60	1.35	2.27
Twigs and leaves	0.90	1.18	1.48

**Table 3:** Inter-day and intraday precision and accuracy.

Concentration	Inter-Day (n = 5)		Intra-Day (n = 5)	
(µg/mL)	CV (%)	RE (%)	CV (%)	RE (%)
1.56	11.25	−16.16	17.67	−19.36
3.13	13.26	10.72	10.5	−4.64
25.00	9.03	−11.08	14.34	−5.88
100.00	5.06	0.63	10.08	1.42

## Data Availability

Data are contained within the article and Supplementary Materials.

## Supplementary Materials

The following supporting information can be downloaded at: https://www.mdpi.com/article/10.3390/plants15091338/s1, The following is the supplementary information to this article: Table S1: Identification of the main compounds in the ethanolic extracts of the flowers, fruits and twigs/leaves of *Cytisus striatus* from Castelo Branco, Guarda and Bragança by GC-MS (% relative content). Results expressed by factorial analysis of variance (ANOVA); Table S2: Identification of compounds contained in ethanolic extracts of *Cytisus striatus* flowers by GC-MS; Table S3: Identification of compounds contained in ethanolic extracts of *Cytisus striatus* fruits by GC-MS; Table S4: Identification of compounds contained in ethanolic extracts of *Cytisus striatus* twigs/leaves by GC-MS; Table S5: LC-MS identification of compounds contained in ethanolic extracts of *Cytisus striatus* at flowers, fruit, and twigs/leaves.
