# Supplementary material for: Chemical Characterisation of Cytisus striatus: A Multi-Technique Approach Using GC-MS, LC-HRMS/MS, NIR, and FT-RAMAN
Source: Plants (Basel). 2026 Apr 28;15(9):1338. doi: 10.3390/plants15091338 (PMC13165026; doi:10.3390/plants15091338)
Supplement: Supplementary file 1 [file plants-15-01338-s001.zip › plants-4195796-supplementary.pdf]

SUPPLEMENTARY MATERIAL

Chemical Characterisation of *Cytisus striatus*: A Multi-Technique Approach Using GC-MS, LC-HRMS/MS, NIR, and FT-RAMAN

Débora Caramelo [1], Tiago A. Fernandes [2,3], Eugenia Gallardo [4,5], Ofélia Anjos [1,6,*] and Jorge Gominho [1]

1 Centro de Estudos Florestais (CEF), Laboratório Associado TERRA, Instituto Superior de Agronomia, Universidade de Lisboa, 1349-017 Lisboa, Portugal; dbrcaramelo@gmail.com (D.C.); jgominho@isa.ulisboa.pt (J.G.)
2 Departamento de Ciências e Tecnologia (DCeT), Universidade Aberta, 1000-013 Lisboa, Portugal; tiago.fernandes@uab.pt
3 MINDlab—Molecular Design & Innovation Laboratory, Centro de Química Estrutural, Institute of Molecular Sciences, Departamento de Engenharia Química, Instituto Superior Técnico, Universidade de Lisboa, 1049-001 Lisboa, Portugal
4 RISE-Health, Departamento de Ciências Médicas, Faculdade de Ciências da Saúde, Universidade da Beira Interior, 6200-506 Covilhã, Portugal; egallardo@fcsaude.ubi.pt
5 Laboratório de Fármaco-Toxicologia—UBIMedical, Universidade da Beira Interior, 6200-284 Covilhã, Portugal
6 Centre for Natural Resources, Environmental and Society, Polytechnic University of Castelo Branco, (CERNAS-IPCB), 6001-909 Castelo Branco, Portugal
* Correspondence: ofelia@ipcb.pt; Tel.: +35-272339900

Table S1: Identification of the main compounds in the ethanolic extracts of the flowers, fruits and twigs/leaves of *Cytisus striatus* from Castelo Branco, Guarda and Bragança by GC-MS (% relative content). Results expressed by factorial analysis of variance (ANOVA).

**Code[1]**	**Compound**	**RT (min)**	**Molecular formula**	**R. Match (%)**	**Relative Content (%)**									**Variance Origin**			
					FCB	FG	FB	FrCB	FrG	FrB	TLCB	TLG	TLB	L	P	LxP	Error
**3**	ethyl hydrogen malonate	8.55	$C_5H_8O_4$	90.5	0.42 ± 0.0007^{bc}	0.37 ± 0.0004^{b}	0.22 ± 0.001^{a}						0.53 ± 0.002^{c}	4.8 (*)	33.3 (***)	54.8 (***)	7.1 (***)
**5**	phenylethyl alcohol	8.89	$C_6H_{10}O$	92.4	0.53 ± 0.0004^{a}	0.44 ± 0.001^{a}	0.42 ±0.002^{a}	0.54 ± 0.0008^{a}						14.6 (***)	53.4 (***)	26.6 (***)	5.4 (***)
**6**	4*H*-Pyran-4-one, 2,3-dihydro-3,5-dihydroxy-6-methyl-	9.51	$C_6H_8O_4$	91.8	2.17 ± 0.0006^{c}	3.82 ± 0.002^{d}	3.43 ± 0.011^{d}	0.10 ± 0.0001^{a}	0.23 ± 0.0001^{a}	0.55 ± 0.0002^{b}	0.57 ± 0.0003^{b}	0.78 ± 0.001^{b}	0.83 ± 0.0004^{b}	4.6 (*)	83.5 (***)	6.8 (*)	5.1 (***)
**7**	benzoic acid	9.93	$C_7H_6O_2$	90.1	0.37 ± 0.0001^{a}	0.68 ± 0.0004^{b}	0.33 ± 0.011^{a}	0.32 ± 0.0001^{a}		0.29 ± 0.0001^{a}				-0.2	59.3 (***)	37.7 (***)	3.2 (***)
**9**	benzofuran, 2,3-dihydro	10.99	C_8H_8O	93.3	2.51 ± 0.003^{d}	2.71 ± 0.002^{d}	1.65 ± 0.006^{c}				0.18 ± 0.0001^{a}	0.60 ± 0.001^{b}	0.43 ± 0.003^{ab}	1.9 (*)	86.7 (***)	6.9 (**)	4.5 (***)
**10**	5-hydroxymethylfurfural	11.18	$C_6H_6O_3$	92.6	0.32 ± 0.001^{a}	1.37 ± 0.002^{b}	5.86 ± 0.022^{c}						0.52 ± 0.0001^{a}	17.8 (***)	30.9 (***)	42.4 (***)	8.8 (***)
**13**	2-methoxy-4-vinylphenol	12.90	$C_9H_{10}O_2$	95.5	0.69 ± 0.001^{b}	0.96 ± 0.0002^{bc}	1.36 ± 0.004^{c}	0.18 ± 0.0001^{a}	0.16 ± 0.0001^{a}	0.27 ± 0.0001^{a}	2.09 ± 0.0007^{d}	4.00 ± 0.006^{e}	2.45 ± 0.001^{d}	5.1 (***)	76.2 (***)	15.7 (***)	3.0 (***)
**14**	eugenol	13.73	$C_{10}H_{12}O_2$	93.7	0.14 ± 0.0001^{a}							0.21 ± 0.0002^{b}	0.43 ± 0.0007^{c}	6.5 (***)	32.5 (***)	59.3 (***)	1.7 (***)

**19**	Dodecanoic acid	17.57	$C_{12}H_{24}O_2$	92.0	0.47 ± 0.0003^{c}	0.43 ± 0.0002^{bc}	0.31 ± 0.001^{a}				0.43 ± 0.0003^{bc}	0.33 ± 0.001^{ab}	0.47 ± 0.0009^{c}	0.4	85.9 (***)	7.7 (*)	6.1 (***)
**31**	Pentadecanoic acid	24.29	$C_{15}H_{30}O_2$	80.7			0.63 ± 0.003^{b}	0.11 ± 0.0001^{a}						13.7 (***)	13.7 (***)	59.9 (***)	12.6 (***)
**36**	Palmitic acid	26.53	$C_{16}H_{32}O_2$	93.7	19.81 ± 0.005^{e}	19.16 ± 0.003^{de}	12.60 ± 0.048^{c}	16.27 ± 0.027^{d}	9.38 ± 0.0007^{b}	16.11 ± 0.003^{d}	5.17 ± 0.002^{a}	9.38 ± 0.001		6.4 (***)	76.5 (***)	13.1 (***)	4.0 (***)
**37**	*trans*-sinapyl alcohol	27.02	$C_{11}H_{14}O_4$	84.3				0.92 ± 0.001^{c}	0.65 ± 0.0002^{b}		1.25 ± 0.001^{d}	0.88 ± 0.0005^{c}	0.46 ± 0.0006^{a}	24.3 (***)	55.1 (***)	19.3 (***)	1.4 (***)
**44**	Phytol	29.30	$C_{20}H_{40}O$	89.6	0.11 ± 0.00003	0.11 ± 0.00005	0.14 ± 0.0007				2.37 ± 0.0007^{a}	2.30 ± 0.003^{a}		12.5 (***)	47.6 (***)	39.1 (***)	0.8 (***)
**46**	Linoleic acid	29.79	$C_{18}H_{32}O_2$	91.4				11.41 ± 0.028^{c}	3.39 ± 0.003^{a}	7.25 ± 0.004^{b}	1.91 ± 0.003^{a}	2.01 ± ^{a}		7.6 (***)	64.1 (***)	24.4 (***)	3.9 (***)
**48**	Linolenic acid	29.92	$C_{18}H_{30}O_2$	92.4	14.71 ± 0.003^{c}	18.00 ± 0.007^{d}	8.57 ± 0.025^{b}				5.13 ± 0.007^{a}	7.29 ± 0.013^{b}	7.59 ± 0.007^{b}	3.7 (***)	79.6 (***)	14.9 (***)	1.8 (***)
**49**	Octadecanoic acid	30.25	$C_{18}H_{36}O_2$	88.7	2.46 ± 0.0007^{c}	2.45 ± 0.0001^{c}	1.60 ± 0.005^{b}	2.72 ± 0.004^{c}	1.38 ± 0.001^{b}	3.64 ± 0.001^{d}	0.57 ± 0.0004^{a}	0.83 ± 0.001^{a}	0.88 ± 0.001^{a}	3.3 (***)	52.7 (***)	40.9 (***)	3.2 (***)
**52**	Lupanine	31.62	$C_{15}H_{24}N_2O$	92.6	0.60 ± 0.0001^{a}	0.68 ± 0.0001^{a}	0.62 ± 0.001^{a}	1.52 ± 0.003^{c}	1.08 ± 0.0004^{b}	0.62 ± 0.0004^{a}	3.34 ± 0.002^{e}	3.70 ± 0.004^{f}	2.02 ± 0.0002^{d}	8.6 (***)	79.0 (***)	10.9 (***)	1.5 (***)
**55**	3β-hydroxylupanine	33.22	$C_{15}H_{24}N_2O_2$	85.4	0.39 ± 0.0001^{c}	0.17 ± 0.0001^{a}	0.47 ± 0.0005^{d}	0.47 ± 0.0005^{d}	0.38 ± 0.0002^{c}	0.33 ± 0.00007^{b}				5.0 (***)	77.2 (***)	16.5 (***)	1.3 (***)
**57**	Oleamide	33.63	$C_{18}H_{35}NO$	90.0	0.86 ± 0.0007^{a}	0.77 ± 0.0004^{a}	0.97 ± 0.003^{a}	1.02 ± 0.001^{a}	5.52 ± 0.001^{b}	8.05 ± 0.0009^{c}	0.90 ± 0.0003^{a}	1.12 ± 0.001^{a}	0.72 ± 0.0004^{a}	12.7 (***)	48.1 (***)	39.0 (***)	0.2 (***)
**62**	2-mono-palmitin	35.96	$C_{19}H_{38}O_4$	87.3	0.40 ± 0.0002^{c}	0.20 ± 0.0002^{b}	0.15 ± 0.0005^{a}			0.41 ± 0.0002^{c}				7.1 (***)	31.4 (***)	60.8 (***)	0.7 (***)
**63**	Homopterocarpin	36.12	$C_{17}H_{16}O_4$	87.8				13.52 ± 0.02^{b}	25.10 ± 0.009^{c}	12.59 ± 0.002^{b}		3.10 ± 0.004^{a}	2.37 ± 0.0002^{a}	6.5 (***)	79.6 (***)	13.3 (***)	0.5 (***)
**64**	Hydroxylupanine	36.17	$C_{15}H_{24}N_2O_2$	93.2	2.90 ± 0.001^{c}	1.97 ± 0.001^{b}	1.30 ± 0.003^{ab}	4.18 ± 0.009^{d}	0.96 ± 0.008^{a}		2.69 ± 0.002^{c}			67.2 (***)	10.1 (***)	17.0 (***)	5.6 (***)

**70**	3β, 13α-dihydroxylupanine	37.67	$C_{15}H_{24}N_2O_3$	88.7	0.65 ± 0.0003^{b}	0.41 ± 0.0002^{a}	0.56 ± 0.001^{b}	1.27 ± 0.002^{c}	0.51 ± 0.001^{ab}	0.75 ± 0.0003^{b}	0.66 ± 0.0005^{b}			38.6 (***)	45.0 (***)	13.9 (***)	2.6 (***)
**72**	Chrysin	38.63	$C_{15}H_{10}O_4$	90.5	12.92 ± 0.009^{ab}	11.01 ± 0.002^{a}	7.51 ± 0.021^{a}	17.02 ± 0.02^{b}	18.76 ± 0.015^{bc}	19.86 ± 0.003^{c}	32.75 ± 0.016^{d}	23.73 ± 0.030^{c}	18.01 ± 0.010^{bc}	9.8 (***)	62.7 (***)	24.3 (***)	3.2 (***)
**76**	(±)-α-tocopherol	45.47	$C_{29}H_{50}O_2$	94.0	1.69 ± 0.0002^{d}	0.30 ± 0.0001^{a}	1.11 ± 0.003^{c}				0.73 ± 0.0006^{b}	1.32 ± 0.002^{c}	1.20 ± 0.00002^{c}	2.8 (***)	55.8 (***)	38.9 (***)	2.4 (***)
**78**	Stigmasterol	48.16	$C_{29}H_{48}O$	86.5	0.43 ± 0.0003^{a}	0.54 ± 0.0002^{a}		0.45 ± 0.003^{a}	0.52 ± 0.0003^{a}	0.60 ± 0.0003^{a}	0.43 ± 0.001^{a}	0.54 ± 0.001^{a}	0.45 ± 0.00003^{a}	11.4 (*)	14.5 (*)	52.5 (**)	21.6 (*)
**79**	*β*-sitosterol	49.66	$C_{29}H_{50}O$	94.0	3.93 ± 0.0005^{c}	3.31 ± 0.001^{bc}	3.33 ± 0.010^{bc}	3.08 ± 0.004^{ab}	3.30 ± 0.001^{bc}	3.31 ± 0.001^{bc}	2.94 ± 0.002^{ab}	2.39 ± 0.004^{a}	2.54 ± 0.002^{a}	2.2	44.4 (***)	15.9	37.5 (***)
**80**	*β*-Amyrin	50.46	$C_{30}H_{50}O$	89.0	0.29 ± 0.00002^{a}		0.32 ± 0.0004^{a}				0.71 ± 0.001^{b}	2.17 ± 0.002^{c}	6.52 ± 0.002^{d}	14.8 (***)	43.1 (***)	41.9 (***)	0.2 (***)

[1]Code of chemical compound in the Heat-map; FCB, flowers from Castelo Branco; FG, flowers from Guarda; FB, flowers from Bragança; FrCB, fruits from Castelo Branco; FrG, fruits from Guarda; FrB, fruits from Bragança; TLCB, twigs/leaves from Castelo Branco; TLG, twigs/leaves from Guarda; TLB, twigs/leaves from Bragança; L, local; P, plant part; LxP – ANOVA interaction between L and P. Mean values with the same letter in a row are not statistically different, $p > 0.05$; * $0.01 < p < 0.05$; ** $0.001 < p < 0.01$; *** $p < 0.001$. Different letters in the same line denote significant differences between flowers, fruits and twigs/leaves of *C. striatus* by the LSD test ($p < 0.05$) for each analytical determination.

Table S2: Identification of compounds contained in ethanolic extracts of *Cytisus striatus* flowers by GC-MS.

**Code**[1]	**Compound**	**RT (min)**	**Molecular structure**	**R. Match (%)**	**Relative Content (%)**		
					**FCB**	**FG**	**FB**
**2**	furaneol	7.73	$C_6H_8O_3$	92.0	0.08 ± 0.0002^{a}	0.19 ± 0.0001^{c}	0.15 ± 0.001^{b}
**8**	hydroxymaltol	10.34	$C_6H_6O_4$	85.4			0.21 ± 0.0009

**11**	1,2,3-propanetriol, 1-acetate	11.50	$C_5H_{10}O_4$	87.8	0.14 ± 0.0004[a]	0.33 ± 0.0005[b]	0.59 ± 0.002[c]
**12**	benzeneacetic acid	11.58	$C_8H_8O_2$	91.6	0.17 ± 0.00004		
**21**	3-benzyl-4-chloro-1,2,3-triazole 1-oxide	17.78	$C_9H_8ClN_3O$	85.2	0.18 ± 0.0001[a]	0.36 ± 0.0004[b]	
**24**	Tridecanoic acid	19.05	$C_{13}H_{26}O_2$	86.0	0.31 ± 0.0002[b]	0.24 ± 0.0001[a]	0.21 ± 0.0009[a]
**27**	Zingerone	19.69	$C_{11}H_{14}O_3$	90.5			0.27 ± 0.0010
**32**	Ferulic acid	24.50	$C_{10}H_{10}O_4$	80.6			0.50 ± 0.0015
**34**	Hexadecanoic acid, methyl ester	25.68	$C_{17}H_{34}O_2$	86.5			0.16 ± 0.0008
**38**	Hexadecanoic acid, ethyl ester	27.05	$C_{18}H_{36}O_2$	88.1	0.47 ± 0.0003[c]	0.31 ± 0.0003[b]	0.17 ± 0.001[a]
**39**	Heptadecanoic acid	27.63	$C_{17}H_{34}O_2$	84.6	0.69 ± 0.0002[b]	0.44 ± 0.0003[a]	0.46 ± 0.002[a]
**42**	Linolenic acid, methyl ester	29.08	$C_{19}H_{32}O_2$	87.8		0.32 ± 0.001[a]	0.14 ± 0.0007[a]
**45**	Tetrahydrorhombifoline	29.48	$C_{15}H_{24}N_2O$	82.8	0.11 ± 0.0001		
**53**	Benzoyl β-d-glucoside	31.91	$C_{13}H_{16}O_7$	88.7	1.48 ± 0.0003[b]	2.28 ± 0.0001[c]	0.37 ± 0.0009[a]
**54**	Pentacosane	32.58	$C_{25}H_{52}$	91.3	1.18 ± 0.009[a]	3.15 ± 0.001[b]	2.38 ± 0.010[b]
**56**	Benzene, 1-1'-(1,2-dimethyl-1,2-ethanediyl) bis-, (R*,S*)	33.46	$C_{16}H_{18}$	92.4	10.13 ± 0.003[c]	5.01 ± 0.001[b]	3.41 ± 0.010[a]
**59**	Hexacosane	34.22	$C_{26}H_{54}$	88.0		0.17 ± 0.0002[b]	0.14 ± 0.0006[a]
**60**	Phenethyl tetradecanoate	35.39	$C_{22}H_{36}O_2$	92.4	0.15 ± 0.0002[b]	0.17 ± 0.00005[b]	0.09 ± 0.0004[a]
**61**	Octacosane	35.81	$C_{28}H_{58}$	86.5	0.23 ± 0.0001[a]	0.86 ± 0.0002[c]	0.49 ± 0.002[b]
**65**	Oxalic acid, 2-phenylethyl tetradecyl ester	36.39	$C_{24}H_{46}O_4$	84.8	0.10 ± 0.00004[c]	0.08 ± 0.00002[b]	0.06 ± 0.0003[a]

**68**	Butanoic acid, 3-methyl-,2-methoxy-4-(2-propenyl)phenyl ester	37.14	$C_{15}H_{20}O_3$	85.2	0.39 ± 0.0002		
**74**	Phenethyl stearate	41.45	$C_{26}H_{44}O_2$	86.0	0.25 ± 0.0001^a	0.33 ± 0.0001^b	
**75**	Hentriacontane	41.58	$C_{31}H_{64}$	85.5		0.30 ± 0.0001^b	0.12 ± 0.001^a

[1]Code of chemical compound in the Heat-map; FCB, flowers from Castelo Branco; FG, flowers from Guarda; FB, flowers from Bragança; L, local; P, plant part; LxP – ANOVA interaction between L and P. Mean values with the same letter in a row are not statistically different, $p > 0.05$; * $0.01 < p < 0.05$; ** $0.001 < p < 0.01$; *** $p < 0.001$. Different letters in the same line denote significant differences between flowers, fruits, and twigs/leaves of *C. striatus* by the LSD test ($p < 0.05$) for each analytical determination.

Table S3: Identification of compounds contained in ethanolic extracts of *Cytisus striatus* fruits by GC-MS.

**Code[1]**	**Compound**	**RT (min)**	**Molecular structure**	**R. Match (%)**	**Relative Content (%)**		
					**FrCB**	**FrG**	**FrB**
**1**	3-methoxy pyridine	6.60	C_6H_7NO	89.6	0.17 ± 0.0001		
**4**	maltol	8.86	$C_6H_6O_3$	93.8		0.43 ± 0.0001^a	0.69 ± 0.000^b
**15**	tetradecane	14.44	$C_{14}H_{30}$	88.0	0.08 ± 0.0004^a	0.15 ± 0.0002^b	
**17**	*β*-D-glucopyranose, 1,6-anhydro	15.97	$C_6H_{10}O_5$	88.9	0.54 ± 0.0002		
**22**	*β*-D-glucopyranoside, methyl	18.06	$C_7H_{14}O_6$	86.0		0.98 ± 0.0006	
**23**	Hexadecane	18.45	$C_{16}H_{34}$	88.7			0.18 ± 0.0002
**25**	ethyl, *α*-D-glucopyranoside	19.12	$C_8H_{16}O_6$	86.9		5.23 ± 0.005^b	1.19 ± 0.0007^a
**28**	Tetradecanoic acid	22.14	$C_{14}H_{28}O_2$	86.9	0.19 ± 0.0005^a		0.20 ± 0.00003^a
**29**	*t*-butylhydroquinone	23.29	$C_{10}H_{14}O_2$	80.3	0.48 ± 0.0004		

**30**	*N*-acetyltyramine	23.83	$C_{10}H_{13}NO_2$	87.3		0.38 ± 0.0005^{a}	0.56 ± 0.0005^{b}
**35**	Palmitoleic acid	25.98	$C_{16}H_{30}O_2$	90.3	0.54 ± 0.001^{b}	0.22 ± 0.0001^{a}	0.59 ± 0.0002^{b}
**40**	9,11-octadecenoic acid, methyl ester	28.96	$C_{19}H_{36}O_2$	80.9	0.11 ± 0.0003		
**41**	13-octadecanoic acid, methyl ester	29.06	$C_{19}H_{36}O_2$	89.0			0.13 ± 0.0001
**43**	Methyl oleate	29.08	$C_{19}H_{36}O_2$	84.3	0.19 ± 0.0005		
**47**	*cis*-vaccenic acid	29.90	$C_{18}H_{34}O_2$	90.3	10.35 ± 0.004^{c}	4.96 ± 0.001^{a}	9.68 ± 0.002^{b}
**50**	Hexadecanamide	30.51	$C_{16}H_{33}NO$	85.6		0.38 ± 0.0001	
**51**	Octadecanoic acid, ethyl ester	30.77	$C_{20}H_{40}O_2$	80.2			0.13 ± 0.001
**58**	Octadecanamide	34.03	$C_{18}H_{37}NO$	90.1			0.21 ± 0.00005
**67**	Medicarpin	36.98	$C_{16}H_{14}O_4$	93.5	0.64 ± 0.001^{a}	0.81 ± 0.001^{b}	
**71**	Pterocarpin	37.84	$C_{17}H_{14}O_5$	87.6	3.36 ± 0.006^{b}	5.67 ± 0.001^{c}	2.22 ± 0.0004^{a}
**77**	Campesterol	47.35	$C_{28}H_{48}O$	84.9			0.93 ± 0.002

[1]Code of chemical compound in the Heat-map; FrCB, fruits from Castelo Branco; FrG, fruits from Guarda; FrB, fruits from Bragança; L, local; P, plant part; LxP – ANOVA interaction between L and P. Mean values with the same letter in a row are not statistically different, p > 0.05; * 0.01 < p < 0.05; ** 0.001< p < 0.01; *** p < 0.001. Different letters in the same line denote significant differences between flowers, fruits, and twigs/leaves of *C. striatus* by the LSD test (p < 0.05) for each analytical determination.

Table S4: Identification of compounds contained in ethanolic extracts of *Cytisus striatus* twigs/leaves by GC-MS.

**Code[1]**	**Compound**	**RT (min)**	**Molecular structure**	**R. Match (%)**	**Relative Content (%)**		
					**TLCB**	**TLFG**	**TLFB**
**16**	sucrose	15.34	$C_{12}H_{22}O_{11}$	84.0	0.84 ± 0.002		

**18**	apocynin	16.09	$C_9H_{10}O_3$	89.0	1.09 ± 0.0005[a]	2.93 ± 0.006[c]	1.79 ± 0.0006[b]
**20**	Phenol, 4-ethenyl-2,6-dimethoxy	17.74	$C_{10}H_{14}O_3$	94.9	2.29 ± 0.001[c]	1.96 ± 0.004[b]	1.48 ± 0.003[a]
**26**	Benzoic acid, 3,4-dimethoxy	19.37	$C_9H_{10}O_4$	90.4	0.62 ± 0.001		
**33**	Neophytadiene	24.76	$C_{20}H_{38}$	89.7	0.53 ± 0.001		
**66**	13α-acetoxylupanine	36.60	$C_{25}H_{42}N_2O_3$	85.7	0.38 ± 0.0003[b]	0.30 ± 0.0003[a]	
**69**	Phenol, 2-methoxy-4-(1-propenyl)-, acetate	37.62	$C_{12}H_{14}O_3$	85.8			0.29 ± 0.0002
**73**	Squalene	40.65	$C_{30}H_{50}$	89.7	0.66 ± 0.0002[b]	0.57 ± 0.0004[a]	0.77 ± 0.0005[c]
**81**	Germanicol	50.67	$C_{30}H_{50}O$	85.4			0.99 ± 0.0002
**82**	Lupeol	51.81	$C_{30}H_{50}O$	89.0	7.17 ± 0.007[b]	3.67 ± 0.003[a]	3.34 ± 0.0008[a]

[1]Code of chemical compound in the Heat-map; TLCB, twigs/leaves from Castelo Branco; TLG, twigs/leaves from Guarda; TLB, twigs/leaves from Bragança; L, local; P, plant part; LxP – ANOVA interaction between L and P. Mean values with the same letter in a row are not statistically different, $p > 0.05$; * $0.01 < p < 0.05$; ** $0.001 < p < 0.01$; *** $p < 0.001$. Different letters in the same line denote significant differences between flowers, fruits and twigs/leaves of *C. striatus* by the LSD test ($p < 0.05$) for each analytical determination.

Table S5: LC-MS identification of compounds contained in ethanolic extracts of *Cytisus striatus* at flowers, fruit, and twigs/leaves.

Code	Compound	RT (min)	Molecular formula	Sample								
				FCB	FG	FB	FrCB	FrG	FrB	TLCB	TLG	TLB
**83**	Chrysin	10.6	$C_{15}H_{10}O_4$	+	+	+	+	+	+	+	+	+
**84**	Apigenin	9.9	$C_{15}H_{10}O_5$	+	+	+	+	+	+	+	+	+
**85**	Chrysin 7-(4″-acetylglucoside)	12.2	$C_{23}H_{22}O_{10}$	+	+	+	+	+	+	+	+	+
**86**	Apigenin-7-glucoside	9.1	$C_{21}H_{20}O_{10}$	+	+	+	+	+	+	+	+	+
**87**	Oroxylin A (5,7-dihydroxy-6-methoxyflavone)	10.1	$C_{16}H_{12}O_5$	–	–	–	+	+*	+*	–	–	–
**88**	Kaempferol	9.7	$C_{15}H_{10}O_6$	+	+	+	+	+	+	+	+	+
**89**	Quercetin 3-galactoside	9.0	$C_{21}H_{20}O_{12}$	+	+	+	–	–	–	–	–	–
**90**	Daidzin (Daidzein 7-*O*-glucoside)	9.8	$C_{21}H_{20}O_9$	+	+	+	+	+	+	+	+	+
**91**	Malonylgenistin	8.7	$C_{24}H_{22}O_{13}$	+*	+*	+*	+	+	+	+	+	+
**92**	Esculetin	9.2	$C_9H_6O_4$	+	+	+	–	–	–	–	–	–
**93**	*trans*-*O*-Coumaric acid 2-glucoside	9.0	$C_{15}H_{18}O_8$	+	+	+	–	–	–	–	–	–
**94**	2-Cinnamoyl-1-galloylglucose	9.4	$C_{22}H_{22}O_{11}$	+	+	+	+	+	+	+	+	+

+ found; – not found; * didn't fragmented; FCB, flowers from Castelo Branco; FG, flowers from Guarda; FB, flowers from Bragança; FrCB, fruits from Castelo Branco; FrG, fruits from Guarda; FrB, fruits from Bragança; TLCB, twigs/leaves from Castelo Branco; TLG, twigs/leaves from Guarda; TLB, twigs/leaves from Bragança
